# Complex physical therapy employing self-adjusting garment (ReadyWrap®) in breast cancer-related lymphedema cases in Brazilian women: a protocol for a randomized controlled trial

**DOI:** 10.1186/s13063-023-07460-4

**Published:** 2023-08-22

**Authors:** Jéssica Malena Pedro da Silva, Raul Denner Duarte Araújo, Francisca Cristina da Silva Santos, Erica Alves Nogueira Fabro, Marcus Vinicius de Mello Pinto, Suzana Sales de Aguiar, Luiz Claudio Santos Thuler, Anke Bergmann

**Affiliations:** 1 Division of Clinical Research and Technological Development, Brazilian National Cancer Institute (INCA), Rio de Janeiro, RJ Brazil; 2 Institute Celulare, Rio de Janeiro, RJ Brazil

**Keywords:** Breast cancer lymphedema, Compression bandages, Rehabilitation medicine, Physiotherapy

## Abstract

**Background:**

Lymphedema is a common complication following breast cancer treatment. The aim of this study is to evaluate the effectiveness of a self-adjusting compression garment (ReadyWrap®) in reducing (phase 1) and maintaining (phase 2) upper limb volume in women presenting breast cancer-related lymphedema.

**Methods:**

This study will comprise a randomized, controlled, single-blind clinical trial concerning women with breast cancer-related lymphedema undergoing treatment at a public cancer treatment reference hospital in the city of Rio de Janeiro, Brazil. The intervention will be carried out by adapting self-dressing versus the standard treatment of compressive bandaging (phase 1) and compressive mesh (phase 2). Both groups will be assessed at the beginning and end of intensive treatment and followed up for up to 12 months to evaluate immediate and late outcomes. Assessments will be carried out by physical upper limb examination (inspection, palpation, volume, dynamometry, and thermography) and questionnaires application to assess patient’s quality of life pertaining to the health, functionality, and symptoms of the affected upper limb, as well adverse effects and adherence to treatment. Data will be analyzed descriptively and analytically through univariate and multiple linear regressions. *P* values < 0.05 will be considered statistically significant.

**Discussion:**

This study will evaluate the effectiveness of a self-adjustable garment (ReadyWrap®) in the treatment of lymphedema secondary to breast cancer in Brazilian women compared to the gold standard treatment for limb volume reduction (phase 1) and maintenance (phase 2) phases comprising, respectively, a compressive bandaging and a compressive mesh. The outcome results will provide data based on both quantitative responses and self-reported participant outcomes. The study will also assess the cost-effectiveness of the ReadyWrap® treatment versus standard care. Finally, we expect to reaffirm one more product/therapy as a treatment for this extremely complex and impactful condition following the data analysis.

**Trial registration:**

NCT04934098 [Clinical trials phase 1]. Registered on June 22, 2021. NCT04881604 [Clinical trials phase 2]. Registered on May 11, 2021.

## Administrative information

Note: the numbers in curly brackets in this protocol refer to SPIRIT checklist item numbers. The order of the items has been modified to group similar items (see http://www.equator-network.org/reporting-guidelines/spirit-2013-statement-defining-standard-protocol-items-for-clinical-trials/).

**Table:** 

Title {1}	**Complex Physical Therapy employing self-adjusting garment (ReadyWrap®) in breast cancer-related lymphedema cases in Brazilian women: A protocol for a randomized clinical trial**
Trial registration {2a and 2b}.	NCT04934098 [Clinical trials phase 1] (registered on June 22, 2021) NCT04881604 [Clinical trials phase 2] (registered on May 11, 2021) CEP/INCA 4611711 [local ethics committee registration approval number]
Protocol version {3}	Version 2 of March 25, 2021
Funding {4}	An agreement was signed among the management of the National Cancer Institute, the institute’s ethics committee, and VENOSAN BRASIL LTDA, a company. The institute provided financial support to students (JMPS, RDDA, and FCSS) enrolled in teaching programs who conducted the research. Furthermore, VENOSAN BRASIL LTDA provided the compressive devices, which were offered free of charge to the research participants.
Author details {5a}	^1^ Division of Clinical Research and Technological Development, Brazilian National Cancer Institute (INCA), Rio de Janeiro, RJ, Brazil ^2^ physiotherapist and researcher at Institute Celulare, Rio de Janeiro, RJ, Brazil
Name and contact information for the trial sponsor {5b}	Anke Bergmann—Brazilian National Cancer Institute (INCA). Address: Rua André Cavalcanti, 37—Centro, Rio de Janeiro—RJ, ZIPCODE 20231–050. Brazil. Phone: + 55 21 3207–6551. E-mail: abergmann@inca.gov.br
Role of sponsor {5c}	Despite the private company providing compression devices free of charge to research participants and the institute offering scholarships to teaching program students, it is important to note that this is an investigator-initiated clinical trial. The funders had no involvement in the study’s design and will not be involved in its execution, data collection, data analysis, data interpretation, or the decision to submit results. Additionally, there was no financial support for the writing of the manuscript.

## Introduction

### Background and rationale {6a}

One of the main late breast cancer treatment-associated complications is lymphedema, a chronic progressive condition that can develop months or years after treatment. This condition generally affects 1 in 5 women, and its risk increases in patients undergoing radiotherapy, affected limb chemotherapy infusion, obese women, patients presenting seroma development after surgery, a greater number of removed lymph nodes, or presenting an advanced disease stage at diagnosis [1–4]. According to a study carried out with a cohort of Brazilian women, the cumulative incidence of lymphedema ranges from 13.5% in 2 years to 41.1% in 10 years [2].

Breast cancer-associated lymphedema results from lymphatic system impairment due to lymphatic fluid drainage obstruction and/or interruption, which leads to the accumulation of protein-rich fluids in interstitial spaces, resulting in local edema in the affected area. This phenomenon can affect the upper limb ipsilateral to the surgery, as well as the chest wall and scapular region [3, 5]. When untreated, edema causes progressive structural skin distortion, such as thickening and loss of elasticity, as well as sensory alterations, and pain, also increasing the risk of infections [5]. Because of this, women with this condition present a variety of physical and psychosocial problems, leading to daily activity limitations and negatively affecting their functionality and quality of life [1, 3, 6, 7].

In this context, lymphedema treatment should be initiated at the first signs, aiming towards a better clinical condition resolution and lower quality of life impacts. The gold standard treatment for lymphedema recommended by the International Society of Lymphology [8] is complex decongestive therapy (CDT), consisting in a combination of manual lymphatic drainage, compression therapy, therapeutic exercises, and skin care [4, 9].

Compression therapy is one of the main therapeutic physiotherapy resources to reduce lymphedema-affected limb volume and improve fibrosclerotic changes. This method consists of two distinct phases, the first aiming to maximize limb volume, traditionally performed with compressive bandages, and the second aiming to maintain limb volume, replacing the bandage by a compressive mesh [5, 9].

Studies have established the use of self-adjusting garments to replace or complement the use of bandages and/or compressive meshes [10, 11]. These garments are defined as a prefabricated system consisting of inelastic material containing self-adjusting compressible components according to the size and shape of the patient’s upper limb, comprising one of their main advantages, as these garments can be adapted by the patients themselves, allowing for adjustments with decreasing limb volumes [11]. Thus, costs associated to individual consultations can be minimized while still maintaining lymphedema control effectiveness. These garments can also be potentially useful for patients who live far from treatment centers or who present functional or social difficulties in commuting.

Few studies, however, have evaluated the effectiveness of compression therapy employing self-adjusting garments compared to compressive bandages in the volume reduction phase (phase 1) and meshes in the volume maintenance phase (phase 2) of upper limbs presenting lymphedema secondary to breast cancer.

### Objectives {7}

The primary aim of this study is to evaluate the effectiveness of a self-adjusting compression garment (ReadyWrap®) in reducing and maintaining upper limb volume in women presenting breast cancer-related lymphedema compared to standard compression therapy. Secondary objectives consist in comparing upper limb volume and tissue changes, muscle strength, functionality, and symptoms, as well as health-related quality of life, while also assessing the incidence of adverse events, treatment adherence, and material and professional costs according to the intervention group.

### Trial design {8}

The trial design is as follows: single-blind, superiority, randomized controlled clinical trial, with a 1:1 allocation ratio for each group. Phase 1 participants cannot be part of phase 2 study and vice versa.

## Methods: participants, interventions, and outcomes

### Study setting {9}

This is a clinical trial developed in a public cancer treatment reference hospital in the city of Rio de Janeiro, Brazil, which will compare the standard lymphedema treatment with a self-adjusting compression garment according to treatment phase (upper limb volume reduction or maintenance).

### Eligibility criteria {10}

Women aged 18 years and older diagnosed with upper limb lymphedema following surgical breast cancer treatment over 6 months previously and evaluated by the hospital’s physiotherapy service will be eligible for the study. The inclusion criteria were defined according to breast cancer-associated lymphedema treatment stages (Table 1):

**Table 1:** Inclusion criteria

Treatment phase	Inclusion criteria
**Phase 1: Volume reduction**	Women with volume changes ≥ 10% or perimetry differences ≥ 3 cm in at least one point compared to the contralateral limb, with an indication of compressive bandaging according to the institutional routine [12]
**Phase 2: Volume maintenance**	Women with stabilized lymphedema with an indication of compressive mesh according to the institutional routine [12]

The following will be excluded from both treatment phases: women presenting the following: lymphedema prior to surgery; lymphedema or bilateral lymphadenectomy; phlogistic signs in the swollen limb; submitted to compressive bandaging in the last 3 months; previous history of allergic reaction to the material employed used in compression therapy; locally, regionally, or remotely active disease; undergoing chemotherapy or radiotherapy; presenting functional upper limb changes prior to the breast cancer diagnosis; presenting heart disease and uncompensated systemic arterial hypertension; psychiatric, mental, and neurological disorders or any cognitive impairment that make answering questionnaires impossible.

### Who will take informed consent? {26a}

Eligible candidates will be invited by sector physiotherapists to participate in the study. If they agree, they will be forwarded to a space reserved for signing the free and informed consent form (FICF), an interview, and initial evaluation with the study researchers.

### Additional consent provisions for collection and use of participant data and biological specimens {26b}

Item not applicable in this research, as biological samples are not used to evaluate the study outcomes.

## Interventions

### Explanation for the choice of comparators {6b}

The control group participants will receive the standard institutional therapy, consisting of the gold standard in lymphedema secondary to breast cancer treatment. Thus, the standard intervention group comprising phase 1 will receive the compressive bandaging treatment, while the standard intervention group of phase 2 will receive the compressive mesh treatment.

### Intervention description {11a}

**Fig. 1:**
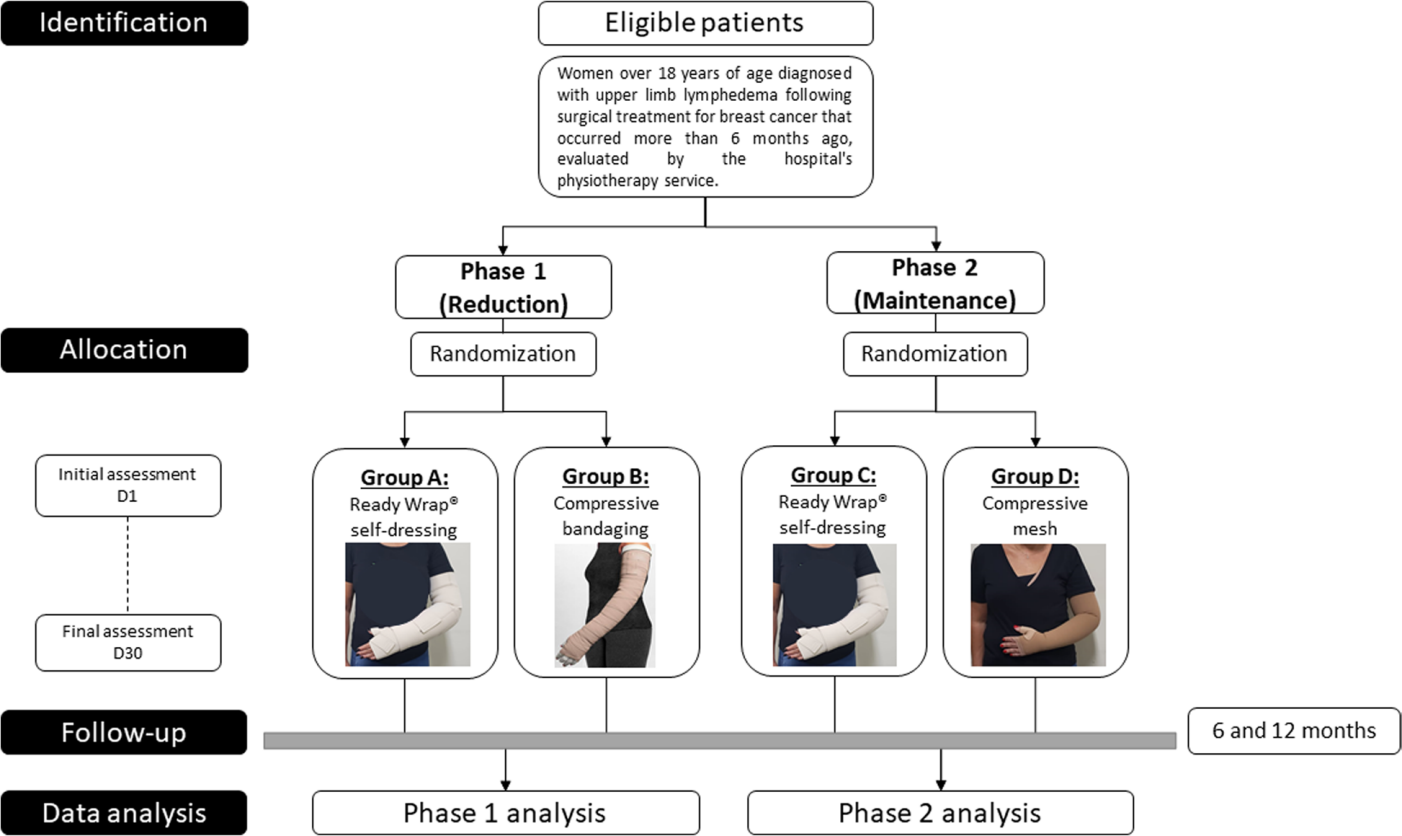
Study schema

The evaluations, interventions, and data collection steps will be carried out by three professional physiotherapists with at least 2 years of experience in compression therapy, all trained and qualified for this purpose. All participants will be guided on the research procedures and will receive a booklet with guidance on performing daily home upper limb exercises, skin care, and daily living activities according to the applied institutional routine [12], which does not include manual lymphatic drainage. Compression therapy will be indicated according to the treatment phase (1 or 2) and intervention group (experimental or control). Participants will be randomly allocated to the following treatment groups (Fig. 1).

### Phase 1 (volume reduction)

#### Group A: Experimental intervention with the ReadyWrap® self-dressing

Participants in this group will receive the ReadyWrap® self-garment (a short-stretch compression wrap, user-adjustable through Velcro straps, which can be made up of up to 2 pieces—arm and hand), according to an ideal size indication derived from affected upper limb perimeter measurements. The garment will be used continuously, maintained for 30 consecutive days, and removed only when bathing. The evaluations will be carried out in a face-to-face consultation on the 1st, 7th, and 30th day after the adaptation period.

A booklet and video were produced exclusively with guidelines on the use of the self-garment and will be made available to participants.

#### Group B: Standard compressive bandaging intervention

This group of participants will receive the standard compressive bandaging treatment for upper limb volume reduction. The compressive bandaging consists of adapting low-elastic bandages placed in layers (multilayer) throughout the affected limb. The upper limb will first be cleaned and hydrated and a tubular mesh will then be worn for protection and for minimizing the risk of allergic reactions. The foams will then be adapted to give the limb a cone shape and soften bone points, contributing to regular pressure distribution. Finally, the bandages will be adapted from the distal to the proximal part. The compressive bandaging will be used continuously for 30 days, changed twice a week in face-to-face consultations. The evaluations will take place on the 1st, 7th, and 30th day after adaptation.

### Phase 2 (volume maintenance)

#### Group C: Experimental intervention with the ReadyWrap® self-dressing garment

The participants in this group will be submitted to a similar treatment as group A, although with the aim of maintaining upper limb volume. The patients will be instructed to remove their garment during nightly sleep, in addition to bath time. After 7 days of self-garment adaptation, the research team will contact the patients by phone to monitor possible adverse events and assess the need for face-to-face consultations. Outpatient follow-up and evaluations in face-to-face consultations will take place on the 1st and 30th day after adaptation.

#### Group D: Standard compressive mesh intervention

This group will receive the standard upper limb volume maintenance treatment employing a compressive mesh that provides 30 to 40 mmHg of pressure and has an elastic Velcro strap for fixation that will be adapted according to ideal size indications following affected upper limb perimetry measurements. The compressive mesh must be continuously used, removing the mesh only for bathing and night sleeping. After 7 days of adaptation, the research team will contact the patients by phone to monitor possible adverse events and assess the need for face-to-face consultations. Outpatient follow-up and evaluations in face-to-face consultations will take place on the 1st and 30th day after adaptation.

Participants in each intervention group will be followed and reassessed 6 and 12 months after study enrollment to verify long-term outcome measures. At the end of this period, the standard compressive mesh will be adapted and provided free of charge according to the applied institutional routine.

### Criteria for discontinuing or modifying allocated interventions {11b}

Treatment suspension before the proposed period will be implemented when side effects occur, such as allergic reactions or poor adaptation to the employed material, as well as limb volume increases greater than 10% of the initial volume, affected upper limb infection and decompensated systemic blood pressure. In the case of any other unforeseen changes, the patient will be evaluated by the health team and any necessary procedures will be adopted.

### Strategies to improve adherence to interventions {11c}

With the aim of improving treatment adherence, the volunteers will receive a therapeutic diary for use in the first 30 days, in which they must notify exercise performance, garment use, and any arm symptoms during treatment daily. In addition, a face-to-face consultation for the phase 1 groups (A and B) and a teleconsultation for the phase 2 groups (C and D) will be performed within 7 days of study inclusion.

### Relevant concomitant care permitted or prohibited during the trial {11d}

It is important to note that any other lymphedema treatment intervention employed by the study participants will not be restricted. Patients should carry out their normal routines and the only interference of this study will be the type of provided compression therapy. Thus, all patients will be instructed to only use the provided compression therapy.

### Provisions for post-trial care {30}

If an increase of over 10% of the initial volume of the upper limb presenting lymphedema is observed during the 30-day reassessment after participant inclusion, compromising patient functionality and/or quality of life, the participant will be directed to the standard treatment offered by the institution’s physiotherapy service. At the end of the study, regardless of the allocation group and results, participants will be directed to lymphedema treatment according to the institutional routine.

## Outcomes {12}

### Primary outcome

#### Lymphedema arm volume

This will be evaluated by determining upper limb perimetry, with reference to the cubital fold, from which points will be measured at 7 cm, 14 cm, and 21 cm below and 7 cm and 14 cm above. The measurements referring to each point will be applied in the trunk cone formula *V* = *h* * (*C*^2^ + *Cc* + *c*^2^)/(π* 12), where *V* is the limb segment volume, *C* and *c* are the circumferences at each end, and *h* is the distance between circles (*C*). This calculation will represent the estimated limb volume [13]. The limb volume percentage reduction will be calculated as (*VI*-*VF*/*VI*)*100, where *VI* comprises the initial volume and *VF* the final volume. The treatment response will be considered effective in phase 1 when an affected upper limb volume reduction of over 10% of the initial volume takes place, while phase 2 therapy success will be linked to volume maintenance or a greater variation of up to 10% of the initial volume of the upper limb presenting lymphedema.

### Secondary outcomes

#### Lymphedema tissue features

These will be determined by means of thermography, a safe, non-invasive imaging method that determines the distribution of body surface temperatures according to physiological and pathological tissue conditions. Inflammation, metabolic subcutaneous tissue changes, and blood supply changes result in temperature gradient changes in the affected area. Thus, different lymphedema stages will be associated to different temperature distribution patterns. A Onepro LT Flir thermographic camera (4800 pixels), with a temperature capture range from – 20 °C to 120 °C will be used to this end. The study participants will be instructed to remain in a standing position, without garments or other trunk and upper limb accessories. Body surface temperatures will be recorded using a thermal camera in the orthostatic position, from the anterior and posterior limb portions, at a 2 m distance. The thermometric images will then be analyzed using the FLIR One***®*** software.

#### Grip strength

This will be evaluated by means of a dynamometer, an instrument widely used to assess handgrip strength. The interviewees will be seated in a chair without armrests with an erect spine, knees flexed at 90°, shoulder positioned in adduction and neutral rotation, elbow flexed at 90°, forearm in half pronation and neutral wrist (or up to 30° extension) [14]. The evaluator supports the weight of the dynamometer and keeps the patient’s arm suspended in the air and the interviewee then performs three bilateral test repetitions. The average of the three measurements will be considered.

#### Health-related QoL

The health-related QoL will be assessed by using two European Organization for Research and Treatment of Cancer (EORTC) questionnaires.

#### Quality of Life Questionnaire Core 30 (EORTC QLQ-C30)

This comprises a specific questionnaire used to assess cancer patients. This instrument comprises 30 questions that derive from functional scales (physical functioning, role functioning, emotional functioning, cognitive functioning, social functioning), symptom/item scales (fatigue, nausea and vomiting, pain, dyspnea, insomnia, appetite loss, constipation, diarrhea, financial difficulties), and global health status/QoL [15].

#### Quality of Life Questionnaire breast cancer-specific (EORTC QLQ-BR 23)

This comprises a specific questionnaire to assess the quality of life of patients presenting breast cancer, consisting of 23 questions divided into functional scales (body image, sexual functional, sexual enjoyment, future perspective) and symptom/item scales (systemic therapy side effects, breast symptoms, arm symptoms, hair loss) [16].

Each scale of both questionnaires scores from 0 to 100. Higher scores in the functional and health-related quality of life (HRQoL) scales indicate a better quality of life, while higher scores in the symptom/item scales translate to a worse quality of life [17]. Both questionnaires have been translated into Portuguese and validated for the Brazilian population [18, 19].

#### Upper limb functionality

Upper limb functionality will be evaluated by the Disabilities of the Arm, Shoulder and Hand Questionnaire (DASH), translated into Portuguese and both validated and adapted for the Brazilian population in 2005 [20]. This questionnaire is widely employed in the evaluation of mild, moderate, and severe shoulder, elbow, and hand disorders. The questionnaire addresses the condition referring to the week before its application and is based on the patient’s ability to perform certain activities, regardless of the used limb. Consisting of 30 questions, the questionnaire evaluates the following domains: physical upper limb function, patient symptomatology, social function, sports practice, music practice, and work activity.

#### Adherence

To assess treatment adherence, participants must fill in a therapeutic diary that will be delivered in the first appointment in which they will be able to notify and grade arm-related symptom intensity through a numerical visual scale (NVS) and write down information they consider relevant concerning the use of compression therapy (such as time of use and adverse events). This resource must be filled out during the 30 days of treatment and given to the therapist during the outpatient consultation.

#### Adverse events

Adverse events will be verified during the first and second treatment phase through therapeutic diary reports and notes regarding the occurrence and intensity of the following symptoms: contact dermatitis, skin infection, uncompensated arterial hypertension, and upper limb volume worsening by 10% of the initial volume, among other unforeseen effects. In addition, all participants will also be provided with a telephone contact that will serve as a communication channel between participants and therapists for reporting adverse events, unpleasant symptoms, and/or questions about the lymphedema treatment, as well as any guidance, when relevant.

#### Treatment costs

The following will be considered in the cost analysis: treatment time, number of sessions, material expenses per patient in each treatment phase, transportation expenses to the hospital from home, and food expenses in the hospital. The patient’s expenses will all be considered, including companion expenses, if any.

### Participant timeline {13}

Figure 1 depicts the patient recruitment, intervention, and evaluation schedule during this study.

### Sample size {14}

Considering the benefits of complex physical therapy when there is a percentage reduction in excess limb volume of 22% to 73% [21], and, for the use of compressive therapy, an estimated response of 33.5% reduction in excess limb volume [22], we stipulated a 35% variation in treatment response between the groups, with an estimated treatment response of 45% in the control group (standard care) and 80% in the intervention group. The sample size was calculated considering a confidence level of 95% with a study power of 80% and a 1:1:1:1 ratio between the intervention groups. Thus, a total population of 120 patients (30 in each group) will be required. To cover possible losses to follow-up and treatment interruption, a total of 20% will be added to the number of participants Therefore, the total sample to be included in this study will be of 144 women (36 in each group).

### Recruitment {15}

Volunteer recruitment will take place at the health institution itself, through post-operative breast cancer follow-up consultations conducted in the physiotherapy sector. The researchers will indicate patients scheduled for consultation who meet the eligibility criteria to participate in the research to the physiotherapy team. If accepted, the patient will be forwarded to the research sector to sign the free and informed consent form and be allocated in a treatment group.

## Assignment of interventions: allocation

### Sequence generation {16a}

The choice between the intervention groups is made from the sequential opening of sealed opaque envelopes, and inside each envelope, there was a card numbered from 01 to 36 for group A or B in phase 1 and for group C or D in phase 2. For each participant who agreed to participate in the study, one of the envelopes was open, depending on the intended lymphedema treatment phase, being randomized between an experimental or control group.

### Concealment mechanism {16b}

Sequentially numbered, opaque, sealed envelopes containing the code that determines participant allocation in the experimental or standard group will be made available according to the treatment phase (phase 1 or phase 2), allocated at a 1:1 ratio in each group. Due to the study characteristics, the allocation will not be blinded and will be revealed to both the participant and the researcher after randomization.

### Implementation {16c}

After recruiting by sector physiotherapists, the participants will sign the free and informed consent form alongside the study researchers, and volunteer randomization in each study arm will be carried out. The study group will be revealed to both the patient and the researcher at the same time.

## Assignment of interventions: blinding

### Who will be blinded {17a}

This comprises a simple blind study in which only the statistical researcher responsible for analyzing the collected data will be blinded.

### Procedure for unblinding if needed {17b}

Due to the study characteristics, both the study participants and the research physiotherapist will be aware of the allocation groups. Consequently, no non-blinding procedure is planned.

## Data collection and management

### Plans for assessment and collection of outcomes {18a}

The data collection will take place through interviews, physical examinations, and medical record analyses. All data will be collected by experienced physiotherapists. The interviews will consist of semi-structured questionnaires produced by the researchers, in addition to questionnaires validated for the Brazilian population. Regarding the physical examination, the volume of the upper limb with lymphedema will be evaluated using perimetry, tissue characteristics using thermography, and muscle strength will be evaluated using dynamometry. These assessment resources will be used for all participants included in the study and performed before and after the initial 30 days of treatment and during follow-up (6 and 12 months). Data regarding oncological treatment and lymphedema treatment history will be obtained from physical and electronic medical records.

### Plans to promote participant retention and complete follow-up {18b}

Study participants will receive the necessary information to understand the evaluation flow and the importance of completing the follow-up, comprising a teleconsultation within 7 days after study inclusion (at the beginning of the treatment) and face-to-face returns for reassessment in 30 days, 6 months, and 1 year. Any study participant can withdraw and leave the study at any time without necessarily explaining the reasons for withdrawal. Thus, whenever problems contacting the participant by phone (call or text message) arise, the institution will ask the institution to send a telegram to the participant’s registered address, instructing them to contact the physiotherapy sector to schedule their appointment.

### Data management {19}

Data will be collected by two physiotherapists who will fill in the information on paper sheets. These will be stored in a safe place for a period of up to 5 years in case there is a need for revision or request by the ethics and research committee. The responsibility for filling in the data in the electronic spreadsheet will be for the same initial collectors who will manually fill. A third member of the study group will review all the data in the electronic database, checking with the paper forms, before exporting to Statistical Package for the Social Science (SPSS).

### Confidentiality {27}

All patients received a random identification number to guaranteeing anonymity. The information collected on paper forms will be stored in a safe place at the Institution’s Clinical Research Division for a period of up to 5 years, in case there is a need for review or request by the ethics and research committee. The data stored in an electronic spreadsheet will be under the responsibility of the main author.

### Plans for collection, laboratory evaluation, and storage of biological specimens for genetic or molecular analysis in this trial/future use {33}

Not applicable, as no biological material will be collected during this study.

## Statistical methods

### Statistical methods for primary and secondary outcomes {20a}

A descriptive analysis of the sociodemographic and clinical characteristics of the study population will be carried out through the distribution of absolute and relative frequencies for categorical variables and central tendency and dispersion measures for continuous variables. The thermographic analysis will be obtained by the average difference in temperatures of one limb in relation to the other and a cut-off point will be established. Data normality will be calculated using the Kolmogorov–Smirnov test. Group comparisons will be done by using Student’s *t* test or Wilcoxon test for continuous variables and chi-square test for categorical variables. The effect size of the mean outcomes will be calculated using Glass’s delta (considering 0.2 as small, 0.5 as medium, and 0.8 as large effects) [23]. Associations between the variables of interest and the outcome will be performed using univariate linear regression. Variables presenting *p* ≤ 0.20 will be included in the multiple linear regression model employing the stepwise forward method, and those with *p* < 0.05 or with clinical relevance will be maintained in the adjusted model. A significance level of 5% will be accepted, at a confidence interval of 95%. Statistical tests will be performed using the SPSS version 23.0 software.

### Interim analyses {21b}

An initial analysis is planned with the data obtained in the 30-day evaluation after study inclusion of each volunteer for both groups, with the statistical analysis carried out by the blind researcher according to the statistical planning.

### Methods for additional analyses (e.g., subgroup analyses) {20b}

There is no plan for subgroup analyses.

### Methods in analysis to handle protocol non-adherence and any statistical methods to handle missing data {20c}

Outcome assessments will be performed by intention to treat and protocol adherence will be verified.

### Plans to give access to the full protocol, participant level-data and statistical code {31c}

The collected and stored data analyzed in this study may be made available by the main investigator in case of formal request with plausible justification for its use.

## Oversight and monitoring

### Composition of the coordinating center and trial steering committee {5d}

This is a study developed in a single location and with few researchers involved. Therefore, no judging committee is included. However, team support is available, as follows: the principal investigator assumes the supervision of the judging committee and technical responsibility for the physiotherapeutic conducts applied to the study participants and is also responsible in case of adverse effects; the study project coordinator carries out test registrations, coordinates technical visits, guarantees the reports, and assists in data organization and protection; physiotherapist 1 performs reassessments and organizes and protects data quality; physiotherapist 2 identifies potential recruits, signals the sector’s physiotherapy team, receives patients, informs the protocol, advises on the informed consent form, performs evaluations, and ensures participant follow-up according to the applied protocol.

### Composition of the data monitoring committee, its role and reporting structure {21a}

A data monitoring committee was not formed for this study. This decision was made based on the absence of serious adverse effects by the applied treatments, both in the experimental treatment and the control, which is the institution’s standard. In any case, control and notifications by the research physiotherapists involved in the study of possible adverse effects and undesirable symptoms reported by the volunteers will be performed. If unwanted effects resulting from the treatment are observed, the participant will withdraw from the study and the institutional routine will be conducted, depending on the situation, and all necessary medical support will be provided.

### Adverse event reporting and harms {22}

The occurrence and intensity of the following symptoms will be verified during the first and second treatment phases through reports and notes made in the therapeutic diary: contact dermatitis, skin infection, uncompensated arterial hypertension, and worsening of upper limb volume by 10% of the initial volume, among other unforeseen effects. In addition, all participants will also be provided with a telephone contact that will serve as a communication channel between participants and therapists for reporting adverse events, unpleasant symptoms, questions about lymphedema treatment, and guidance, when relevant. Treatment suspension before the proposed period will take place when side effects are observed, such as allergic reaction or poor adaptation to the applied material, increase in upper limb volume by 10% of the initial volume, affected upper limb infection, and decompensated blood pressure. If any other unforeseen change occurs, the case will be evaluated by the health team and the necessary procedures will be adopted.

### Frequency and plans for auditing trial conduct {23}

No audits were planned or contracted for this study.

### Plans for communicating important protocol amendments to relevant parties (e.g., trial participants, ethical committees) {25}

Any and all substantial changes that will affect the conduct of this research project will be communicated to the research ethics committee of the previously registered and approved institution for analysis and any judgment. In case the changes affect the participants in any way, they will also be informed about such changes.

### Dissemination plans {31a}

The results of this study will be disseminated through indexed publications in peer-reviewed scientific journals and will also be presented to health professionals at national and international scientific events and disseminated on social media for the general population.

## Discussion

This study will evaluate the effectiveness of the self-adjusting garment (ReadyWrap®) in the treatment of lymphedema secondary to breast cancer in Brazilian women compared to the gold standard treatment for volume reduction (phase 1) and maintenance (phase 2) comprising a compressive bandaging and a compressive mesh, respectively. Outcome results will provide data based on both quantitative responses and self-reported participant outcomes. The study will also assess the cost-effectiveness of the ReadyWrap® treatment versus standard care. Finally, after the data analysis, we expect to propose another compression modality for the treatment of this extremely complex and impactful condition.

### Strong points

This is the first study to comparatively evaluate the use of a self-adjusting compression garment versus traditional therapeutic compression resources applied to lymphedema treatments in two treatment phases, namely volume reduction and maintenance in the Brazilian population. Furthermore, this study is a pioneer in the use of clinical thermography to assess lymphedema tissue characteristics in affected upper limbs. Finally, the planned follow-up will allow for the observation of both short- and long-term outcomes.

### Limitations

Due to the characteristics of this type of study, this clinical trial is single blinded, as there is no possibility of blinding the participants or therapists.

## Trial status

This study is a version 2 submitted and approved on March 25, 2021, by the local research ethics committee. Patient recruitment began in August 2021 and is estimated to end in June 2023. A total of 115 participants were included to date (May 24, 2023).

## Data Availability

Only the study researchers will have direct access to the final analyzed data. The principal investigator of the study, who is the corresponding author of this article, may authorize data sharing upon request with reasonable justification. All data will maintain participant anonymity.

## Abbreviations

REC: Research ethics committee
INCA: Brazilian National Cancer Institute
QL: Quality of life
SPSS: Statistical Package for the Social Sciences
FICF: Free and informed consent form
EORTC: European Organization for Research and Treatment of Cancer
HRQL: Health-related quality of life
DASH: Disabilities of the Arm, Shoulder and Hand Questionnaire
VI: Initial volume
VF: Final volume
V: Limb segment volume
C: Limb circumferences
H: Distance between circles
EVN: Numeric visual scale
